# The Upregulated Expression of the Citrus *RIN4* Gene in HLB Diseased Citrus Aids *Candidatus* Liberibacter Asiaticus Infection

**DOI:** 10.3390/ijms23136971

**Published:** 2022-06-23

**Authors:** Chunzhen Cheng, Yun Zhong, Bin Wang, Yongyan Zhang, Huan Wu, Nonghui Jiang, Bo Wu, Yuanda Lv, Bo Jiang

**Affiliations:** 1 College of Horticulture, Shanxi Agricultural University, Jinzhong 030801, China; ld0532cheng@126.com (C.C.); wb971220@163.com (B.W.); zhyy0425@126.com (Y.Z.); wuhuan980422@163.com (H.W.); 2 Institute of Fruit Tree Research, Guangdong Academy of Agricultural Sciences, Guangzhou 510640, China; jiangnonghui2002@163.com (N.J.); aragornwubo@163.com (B.W.); jiangbo10086@126.com (B.J.); 3 Key Laboratory of South Subtropical Fruit Biology and Genetic Resource Utilization, Ministry of Agriculture and Rural Affairs, Guangzhou 510640, China; lvyuanda2008@163.com; 4 Guangdong Province Key Laboratory of Tropical and Subtropical Fruit Tree Research, Guangzhou 510640, China

**Keywords:** citrus, *RIN4*, Huanglongbing, disease response, genetic transformation

## Abstract

The citrus industry has been threatened by Huanglongbing (HLB) for over a century. Here, an HLB-induced Arabidopsis *RPM1-interacting protein 4* (*RIN4*) homologous gene was cloned from *Citrus clementina*, and its characteristics and function were analyzed to determine its role during citrus–*Candidatus* Liberibacter asiaticus (*C*Las) interactions. Quantitative real-time PCR showed that *RIN4* was expressed in roots, stems, leaves and flowers, with the greatest expression level in leaves. Its expression was suppressed by gibberellic acid, indole-3-acetic acid, salicylic acid and jasmonic acid treatments, but was induced by abscisic acid and salt treatments, as well as wounding. The transient expression of a RIN4-GFP showed that RIN4 was localized in the cell membrane. *RIN4*-overexpressing transgenic *C. maxima* cv. ‘Shatianyou’ plants were obtained, and some transgenic plants showed greater sensitivity to *C*Las infection and earlier HLB symptoms appearance than non-transgenic controls. Results obtained in this study indicated that the upregulated expression of *RIN4* in HLB diseased citrus may aid *C*Las infection.

## 1. Introduction

Plants developed complex immune mechanisms, including pathogen-associated molecular pattern (PAMP)-triggered immunity (PTI) and effector-triggered immunity (ETI), against pathogens during their long-term competition and coevolution [1,2,3,4,5]. PTI recognizes PAMPs through pattern recognition receptors to activate signal pathways in vivo and induce defense responses to limit pathogen invasion [6,7,8]. ETI is monitored by the resistance (R) proteins and can be activated when the R protein and the pathogen’s Avirulence (Avr) protein coexist and interact. There are three general models for the R-Avr interactions, including direct recognition (receptor–ligand model), indirect recognition (including guard, decoy and bait and switch models) and transcriptional regulatory models [9]. After the recognition of a pathogen effector, a plant induces the hypersensitive response of cells in the infected areas, thus limiting the further expansion and invasion of pathogenic microorganisms [6,7]. 

RIN4, a small cell membrane-localized protein, has been confirmed as a plant immunity regulator that plays important roles in both PTI and ETI [10,11,12,13,14]. In Arabidopsis, the phosphorylation at S141 in RIN4 protein was triggered upon bacterial flagellin perception, which will negatively regulate the PTI responses [14,15]. Consistently, transgenic Arabidopsis plants overexpressing *RIN4* displayed reduced PTI responses and the *RIN4* mutant plant exhibited enhanced PTI responses [15]. Moreover, RIN4 can be targeted and modified by several different bacterial effectors, such as AvrB [16], AvrRpm1 [16], AvrRpt2 [17,18] and others [15,19,20], and can interact with plant RPM1 [16], RPS2 [17,21,22,23] and some other R proteins [24,25,26], thus plays roles in the ETI responses [15]. RIN4 can also interact with many other plant proteins. For example, RIN4 also acts as a positive regulator of the jasmonate-signaling pathway downstream of the MPK4 protein, which promotes disease susceptibility [19]. The overexpression of *non-race-specific disease resistance 1* (*NDR1*) gene would greatly improve the plant’s pathogen resistance [27]. In Arabidopsis, the interaction between RIN4 and NDR1 was also identified [28]. Interactions between RIN4 and two cell membrane H+-ATPases contributed to stomatal opening, which were beneficial to microorganism invasions [25]. The H+-ATPase activity was affected in *RIN4* overexpressing and knockout transgenic plants, and the stomata of *rin4* transgenic plants could not be reopened after *Pseudomonas* infection, thereby, preventing bacterial invasion. GCN4 (general control non-repressible 4), a AAA+-ATPase protein, was identified to have the ability of degrading RIN4, thus plays important roles in regulating stomatal aperture and plant defense responses [29]. Additionally, *RIN4* is expressed in guard cells, indicating that these cells are very important in the plants’ innate immunity. In Arabidopsis, the interaction between RIN4 and the exocyst subunit EXO70B1 was also identified, and transiently expressed RIN4 recruited a defense response- and autophagy-related protein EXO70B1 to the plasma membrane [30,31,32], indicating that RIN4 is very important for plant defense reactions. 

In addition to Arabidopsis, RIN4 or RIN4-like proteins play roles in defense reactions in many other plants [10]. In tomato, *RIN4* overexpression inhibits the function of the avirulence factor AvrRpt2, whereas its down-regulation increases tomato resistance to avirulence factor AvrPto [33]. It was determined that RIN4 is probably related to resistance to potato late blight [34]. RPG1-B, which is similar to Arabidopsis R protein RPM1, requires the involvement of a RIN4-like protein to mediate the resistance to AvrB in soybean [7,12]. In addition, *GmRIN4* genes have been reported to negatively regulate the soybean basal resistance against *Pseudomonas syringae* and oomycete pathogens [12]. Banana *RIN4* expression was found to be greatly suppressed by *Foc*TR4 (*Fusarium oxysporum* f. sp. *cubense* tropical race 4) infection in both banana-wilt resistant and susceptible banana varieties, suggesting that its down-regulation may be related to the Fusarium wilt disease-resistance of banana [35]. In *Brassica rapa*, a *RIN4* gene was identified as one of the 15 hub genes involving in the plant immune responses to *Plasmodiophora brassicae* infection [36]. 

In our previous study, a citrus *RIN4* gene (Ciclev10021726m) was identified to be significantly induced in *Candidatus* Liberibacter asiaticus (*C*Las)-infected red tangerine roots at both 20 days post infection (dpi) and 50 dpi [37]. Given the negative regulatory roles of RIN4 on defense-related processes in many plants, it was inferred that the up-regulation of the *RIN4* gene may function in citrus-*C*Las interactions, and its upregulated expression may be related to the high susceptibility of citrus to HLB. In this study, to confirm the role of RIN4 gene upregulation in HLB diseased citrus plants, we cloned the citrus *RIN4* gene, performed sequence, subcellular localization and expression analyses, constructed *RIN4* overexpression vector and obtained the corresponding transgenic *Citrus maxima* cv. ‘Shatianyou’ plants. An HLB-challenge experiment was then performed on transgenic plants to reveal the role of *RIN4* in citrus responses to *C*Las.

## 2. Results

### 2.1. Cloning and Sequence Analysis of Citrus Clementina RIN4

**Figure 1:**
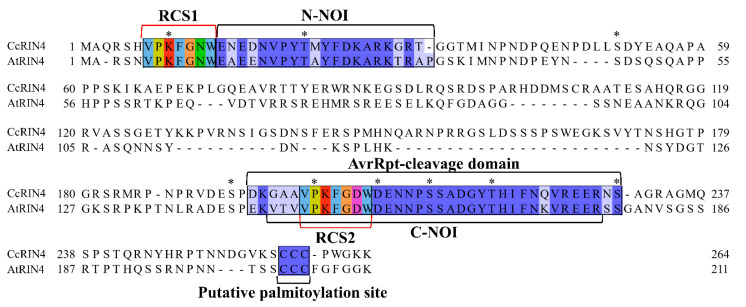
Sequence comparison analysis result of Arabidopsis and *Citrus clementina* RIN4 proteins. RCS 1 and 2: RIN4 cleavage site 1 and 2; the AvrRpt-cleavage domain was predicted by using CDD; *: represents the conserved amino acid residue that might play roles in the posttranscriptional modifications of RIN4. N-NOI and C-NOI represents the N-terminal nitrate induced domain and C-terminal nitrate induced domain, respectively.

A 795 bp long *RIN4* gene was cloned from *C. clementina* [38]. A bioinformatics analysis showed that the putative RIN4 protein consisted of 264 amino acids without a signal peptide or transmembrane structure. Moreover, an AvrRpt-cleavage domain, ranging from the 195–230 amino acid, was predicted using CDD (conserved domains database) (Figure 1). By comparison with Arabidopsis RIN4, the same two AvrRpt2 cleavage sites (RCS1, sequence VPKFGNW and RCS2, sequence VPKFGDW) were found at the N-terminal side of N-NOI (N-terminal nitrate induced) domain and inside the C-NOI (C-terminal NOI) domain, respectively. In addition, a putative palmitoylation site containing three cysteines at C257-C259 was also identified [18]. Additionally, 35 phosphorylation sites (26 on serine (S), 6 on threonine (T) and 3 on tyrosine (Y)) were predicted in the RIN4 protein. What is more, we found that the citrus and Arabidopsis RIN4 proteins shared many conserved amino acid residues, including the K8, T21, S47, S141, P149, D153, S160, S166 and S178 residues in Arabidopsis, which were previously reported to be critical for the transcriptional modifications of Arabidopsis RIN4 [4,14].

**Figure 2:**
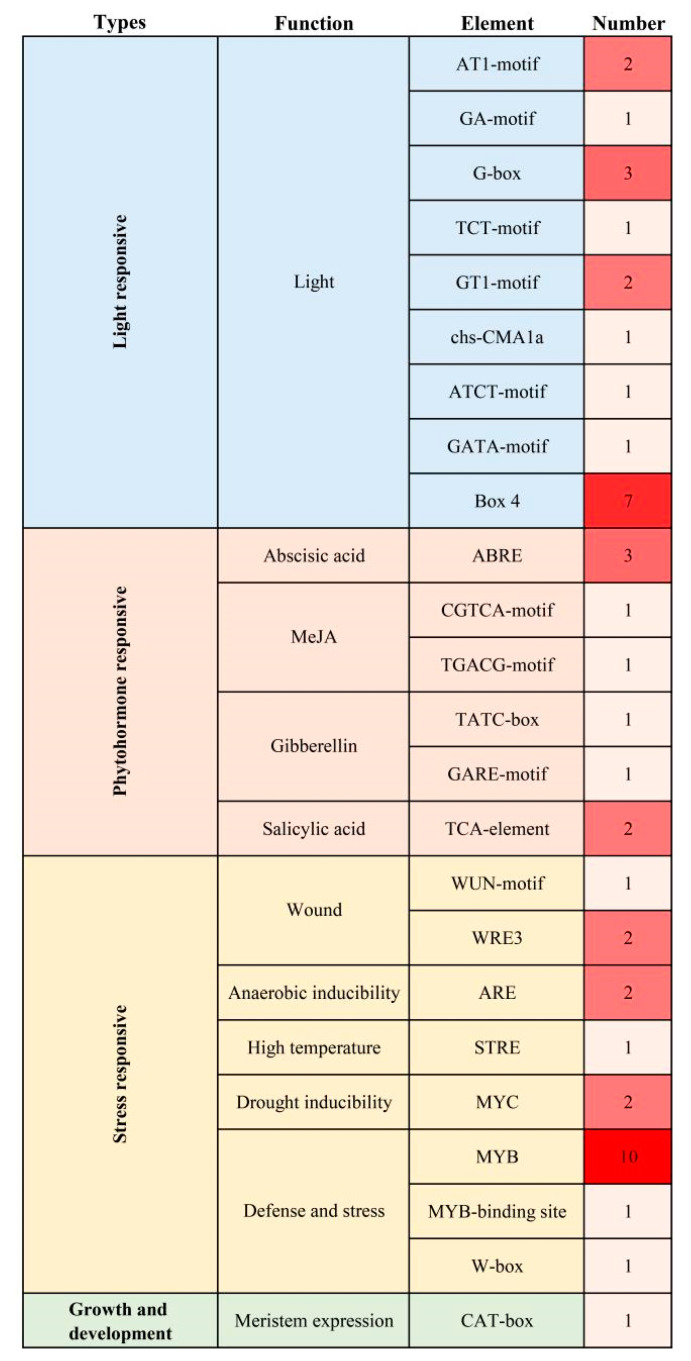
*Cis*-acting elements identified in the *Citrus clementina RIN4* promoter. According to the annotated functions of the identified elements, *cis*-elements were mainly divided into four types, including light responsive, phytohormone responsive, stress responsive and growth and development related. The numbers represent the abundances of identified *cis*-elements in the *RIN4* promoter, and the redder the color, the higher the abundance.

Promoter sequence analysis revealed that the *RIN4* promoter contained several phytohormone responsive elements, including three abscisic acid (ABA) responsive (ABREs), two GA-responsive (TATC-motif and GARE-motif), two SA-responsive (TCA-elements) and two MeJA responsive elements (CGTCA-motif and TGACG-motif), and many stress responsive and light-responsive elements (Figure 2), indicating that *RIN4* may participate in a variety of hormone- and stress-responses.

### 2.2. Subcellular Localization of RIN4

**Figure 3:**
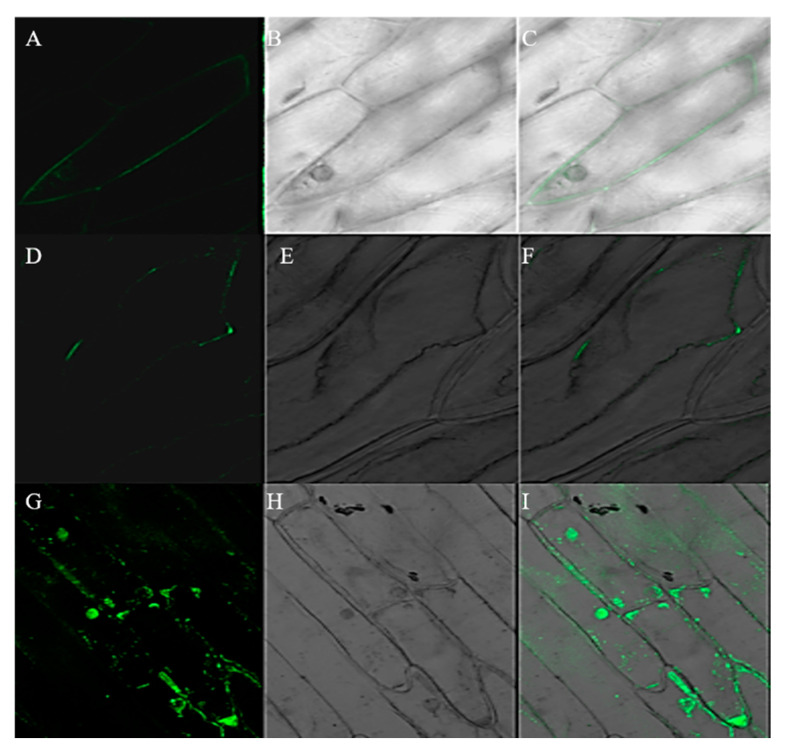
Subcellular localization of the RIN4-GFP protein in onion epidermal cells. (**A**–**C**): before plasmolysis; (**D**–**F**): post plasmolysis; (**G**–**I**): pBEGFP control vector; (**A**,**D**,**G**): fluorescent field; B, (**E**,**H**): bright field; (**C**,**F**,**I**): merged field.

The vector harboring a *RIN4-GFP* fusion gene and the pBEGFP control vector were separately transformed into onion epidermal cells using *Agrobacterium*-mediated transformation method, which resulted in a highly efficient transient expression. As shown in Figure 3, fluorescence signals were found throughout the cells when transformed by pBEGFP control vector, including cell membrane, nuclei and cytoplasm, while in the cells transformed by *RIN4-GFP*, the fluorescence signal was only observed in cell membrane. To further exclude the localization of fluorescence in cell wall, plasmolysis was performed. After plasmolysis, the RIN4 protein was identified to be cell membrane-localized, which is the same to the Arabidopsis RIN4 [10,16,18].

### 2.3. Effect of CLas Infection on RIN4 Expression

**Figure 4:**
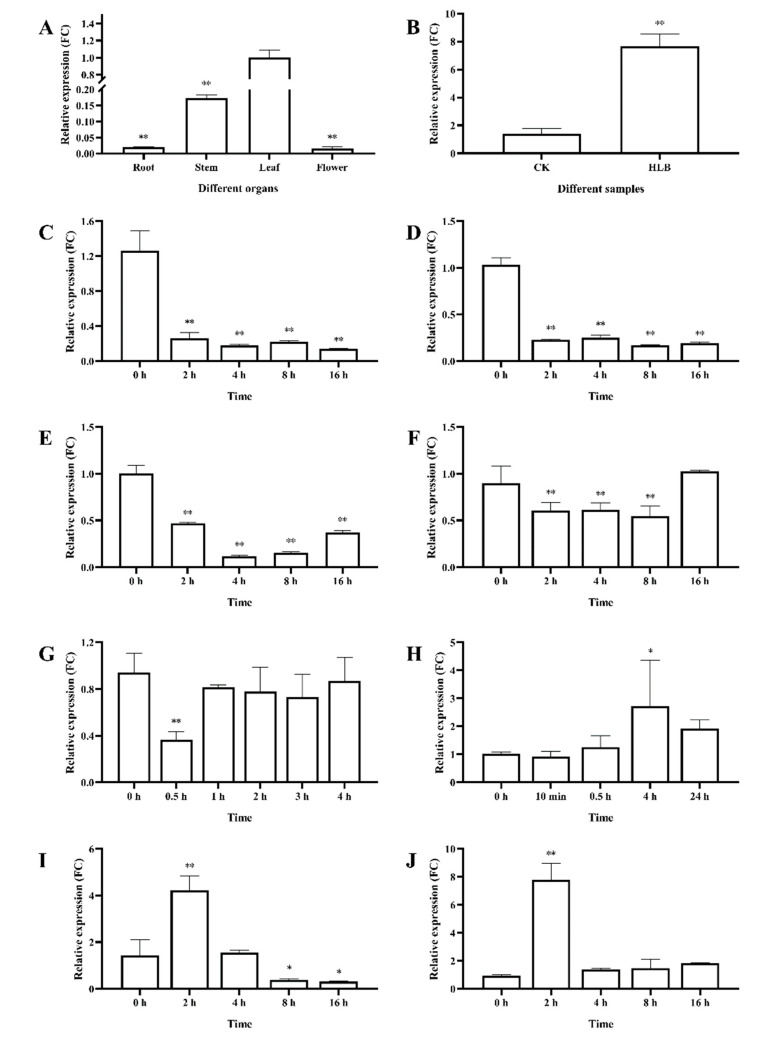
Expression levels of *RIN4* in different organs (**A**), in leaves of HLB infected citrus (**B**) and in leaves under different treatments (**C**–**J**). FC: fold change; (**A**): Different organs; (**B**): *RIN4* expression in leaves of healthy and HLB infected citrus; (**C**): GA treatment; (**D**): IAA treatment; (**E**): JA treatment; (**F**): SA treatment; (**G**): PEG treatment; (**H**): Wound treatment; (**I**): Salt treatment; (**J**): ABA treatment; The results were shown as mean ± standard deviation (SD); *: significant difference identified at *p*-value < 0.05 compared with the control; **: very significant difference identified at *p*-value < 0.01 compared with the control.

Using qRT-PCR, the expression level of *RIN4* in different sweet orange organs was investigated. The highest expression was found in leaf, and the gene’s expression level in stem, root and flower were only 17.3%, 1.9% and 1.6%, respectively, of that in leaf (Figure 4A). We further investigated the relative expression of *RIN4* in HLB infected red tangerine leaf. Result showed that the expression of *RIN4* in the leaves of red tangerine at 2 months after *C*Las inoculation was ~7.6 fold higher than that of the control (Figure 4B), indicating that the expression of *RIN4* was induced by HLB.

### 2.4. RIN4 Expression Levels under Different Phytohormone and Stress Treatments

We further studied the *RIN4* expression patterns under different phytohormone and abiotic stress treatments. GA, IAA and JA very significantly inhibited the *RIN4* expression at all of the tested time points after treatment (Figure 4C–E). SA also very significantly suppressed its expression at 2, 4 and 8 h post treatment, but its expression recovered to the normal level by 16 h (Figure 4F). 

The PEG treatment only very significantly inhibited *RIN4* expression in the early stage (0.5 h after treatment), while later the expression level was similar to that of the control (Figure 4G). Wounding induced *RIN4* expression, which peaked at 4 h after treatment (~2.7 times the level of the control) and then declined (Figure 4H). After 2 h of salt treatment, *RIN4* expression increased 4.2-fold and after 4 h the up-regulation trend decreased (Figure 4I). After 8 h of salt treatment, the expression of *RIN4* was lower than that of the control (Figure 4I). After a 2 h ABA treatment, *RIN4* expression increased by 7.8-fold and then declined, but its expression level was still greater than that of the control (Figure 4J).

### 2.5. Vector Construction and Genetic Transformation Results

To further clarify the role of *RIN4* in citrus-CLas interactions, we constructed *RIN4*-overexpression vector that was transformed into ‘Shatianyou’ pomelo using *Agrobacterium*-mediated genetic transformations. The regenerated shoots were grafted onto annual red tangerine rootstocks (Supplemental Figure S1). After growing to the five to eight-leaf stage, leaf DNA was extracted for PCR confirmation. In total, 16 *RIN4*-overexpression transgenic plants were obtained [38]. After the second round PCR detection confirmation of the graft-propagated transgenic plants, five transgenic lines (named as RIN4-O1~RIN4-O5) were used for further study.

### 2.6. Analysis of RIN4 Expression in Transgenic Plants

**Figure 5:**
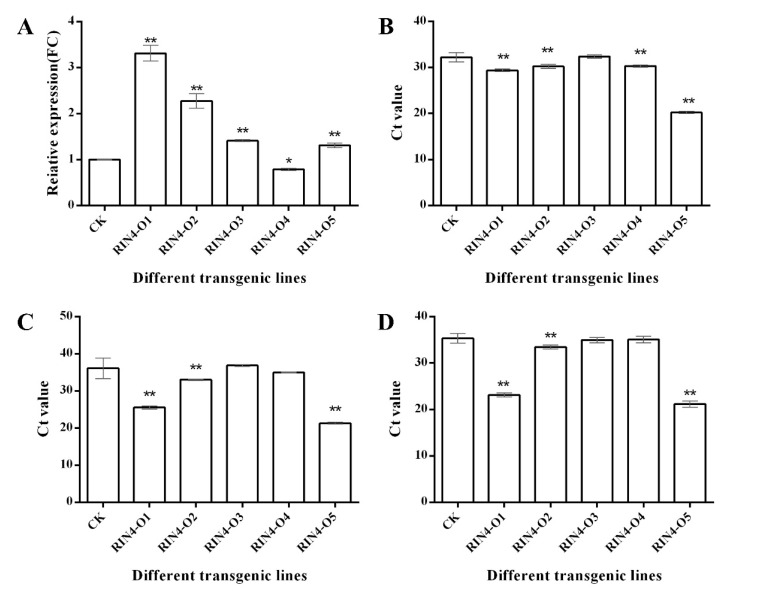
Expression of *RIN4* (**A**) and *C*Las titers (**B**–**D**) in transgenic plants. FC: fold change. The CLas titters in non-transgenic control and *RIN4*-overexpression transgenic pomelo trees were detected at 1, 2 and 3 mpi, respectively (**B**–**D**). The results were shown as average Ct values of three technical repetitions. mpi: months post inoculation. CK: non-transgenic control plants. RIN4-O1~ RIN4-O5: five different *RIN4*-overexpression transgenic lines. The results were shown as mean ± standard deviation (SD). * and ** indicate significant changes at *p* < 0.05 and *p* < 0.01, respectively.

Total RNA of five *RIN4*-overexpression transgenic and five non-transgenic control ‘Shatianyou’ pomelo leaves were extracted. The expression levels of *RIN4* were analyzed by qRT-PCR. Except overexpression line (O) RIN4-O4, the expression levels of *RIN4* in the other four *RIN4*-overexpression transgenic lines were all very significantly greater than that in the control. The greatest expression, approximately 3.5 times greater than that of the control was found in RIN4-O1 (Figure 5A).

### 2.7. Evaluation of HLB Resistance in RIN4-Overexpression Plants

**Figure 6:**
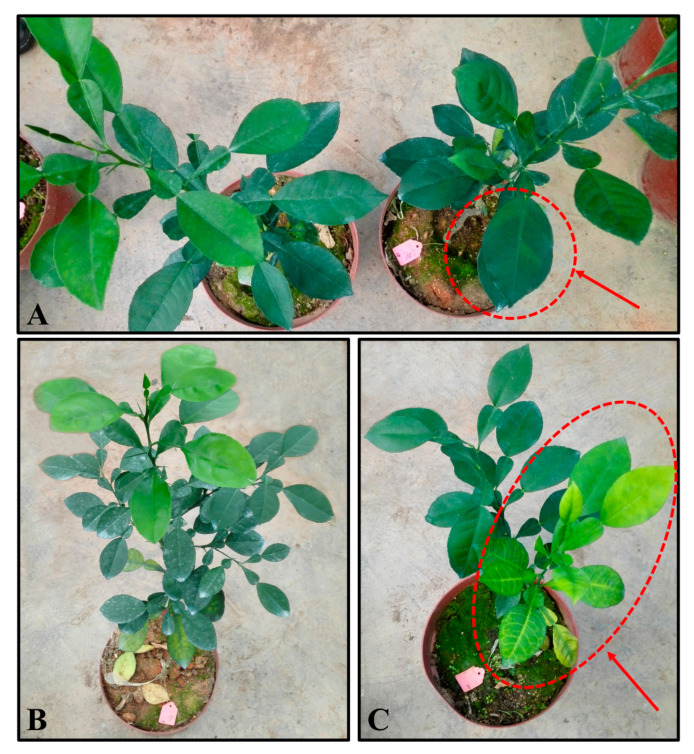
HLB-resistance evaluation of transgenic and non-transgenic control pomelo trees. (**A**): *C*Las-infected non-transgenic tree (left) and *RIN4*-overexpression transgenic RIN4-O5 tree (right) at 2 months post inoculation (mpi). The red arrow indicates the typical HLB mottle leaf symptom. (**B**): *C*Las-free *RIN4*-overexpression transgenic plant; (**C**): *C*Las-infected RIN4-O2 plant showed vein yellowing symptom at 10 mpi.

The transgenic and the non-transgenic control plants were propagated by grafting, and the plants were subjected to the HLB-resistance evaluation when they were ~20 cm tall. HLB could be detected in all transgenic *RIN4*-overexpression and non-transgenic control plants at 1 month post *C*Las inoculation (mpi). At 1~3 mpi, *C*Las titers in the *RIN4*-overexpression and control plants were detected using quantitative Taqman PCR. All of the non-transgenic control plants had Ct values larger than 30 (Figure 5B–D), and the average Ct values of all the non-transgenic plants were 32.2, 36.1 and 35.3 at 1, 2 and 3 mpi, respectively. Noteworthily, the average Ct values of the transgenic RIN4-O1, RIN4-O2 and RIN4-O5 plants were always lower than the non-transgenic controls, indicating that the pathogenic bacteria titters in these transgenic plants were higher than the non-transgenic control plants. The average Ct values of the transgenic RIN4-O5 and RIN4-O1 plants were less than 30 at 1 mpi (20.2 and 29.4, respectively), less than 26 at 2 mpi (21.3 and 25.6, respectively) and less than 24 at 3 mpi (21.2 and 23.1, respectively) (Figure 5). Moreover, the transgenic line RIN4-O5 showed leaf-mottle symptoms at 2 mpi (Figure 6). The Ct value of RIN4-O2 transgenic plant was lower than the non-transgenic plants and it showed vein yellowing at 10 mpi (Figure 5D and Figure 6D); however, the non-transgenic plants did not show obvious HLB diseased symptoms in the meanwhile. These results indicated that the overexpression of the *RIN4* gene contributes to infection by *C*Las and HLB symptom development.

## 3. Discussion

The citrus industry has been threatened by HLB for a long time [39,40]. At present, it is still the most devastating disease of citrus without a cure. What is worse, Ca. Liberibacter can infect all known citrus varieties and their closely related genera [41]. Genetic engineering can enhance plant disease resistance in a relatively short time by overexpressing defense-related genes, including *PRRs* and *Rs* [42,43,44,45,46], or by silencing negative regulator genes. *RIN4* has been confirmed to be a negative regulator of the ETI and PTI pathways in plants [9,47]. *RIN4* overexpression inhibits RPS2-induced hypersensitivity [17], while its degradation resulted in an increased induction of disease responses [33]. In this study, a citrus *RIN4* gene was successfully cloned and its HLB-inducible characteristics were determined using qRT-PCR. A bioinformatics analysis showed that the predicted encoded protein of *RIN4* contained the same RCS1 and RCS2 sequences as Arabidopsis RIN4. As Ca. Liberibacter contain only type I secretion system genes [48,49], the existence of these cleavage sites might serve as targets of Avr proteins form some other pathogens.

As a powerful regulator of plant defense responses [50], posttranscriptional modifications of RIN4 protein have been reported to be key factors influencing the RIN4 function and plant immune system [51]. Compared to Arabidopsis RIN4, citrus RIN4 contains most of the conserved amino acid residues that required for its posttranscriptional modifications, such as the phosphorylation sites T21 [52], S141 and S160 [14,53] and phosphorylation and acetylation site T166 [20], isomerization site P149 [24], ribosylation site D153 [54,55] and acetylation sites K8, S47, S79 and S178 [4,56]. Moreover, in this study, we predicted 35 phosphorylation sites in RIN4, accounting for 13.3% of all amino acids. These phosphorylation sites might be closely related to the phosphorylation modifications of RIN4 during plant immunity [51].

*RIN4* was most highly expressed in leaves, followed by stems. The richness of the light-responsive elements identified in the *RIN4* promoter might be related to its aboveground part high-expression characteristic. The *RIN4* promoter includes several phytohormone-responsive elements, such as GA- and SA-responsive elements. Thus, *RIN4* may participate in a variety of phytohormone- and stress-related responses. Our qRT-PCR result verified that the expression of *RIN4* could be regulated by phytohormone and stress treatments. Treatments that are beneficial to plant growth and development, as well as plant resistance, such as GA, IAA, JA and SA, had inhibitory effects on the *RIN4* expression level. It was reported that *C*Las infections increased the auxins, SAs and JAs levels in the host citrus [57]. Therefore, it was hypothesized that the suppression effects of these phytohormones on *RIN4* expression might be helpful for the plant immunity responses. Moreover, in 2022, Ma et al. reported that foliar gibberellin spray treatment could reduce symptoms of HLB-affected citrus [47], indicating that the GA suppression on *RIN4* expression might play an important role in the gibberellin mediated citrus response to HLB. We also found that treatments that are harmful to plant growth, such as ABA, salt and wounding, had inductive effects on the *RIN4* expression level. Thus, *RIN4* appears to be stress responsive. Noteworthily, the ABA levels in *C*las-infected citrus plants were about 6.1-fold of the healthy controls [57]. In our present study, we found that the *RIN4* expression increased by 7.8-fold after a 2 h ABA treatment, suggesting that the HLB-inducible characteristics of *RIN4* might be mainly related to alterations in ABA levels caused by *C*Las infection. To characterize the role of *RIN4* in citrus responses to *C*Las, a number of *RIN4*-overexpression transgenic plants were obtained through *Agrobacterium*-mediated genetic transformation. Transgenic plants were inoculated with *C*Las for resistance evaluation. Although no significant negative correlation was found among *RIN4* expression level, *C*Las titter and disease symptom severity, the titer of *C*Las in some *RIN4*-overexpression plants (RIN4-O5, RIN4-O1 and RIN4-O2, with significant higher *RIN4* expression than non-transgenic controls) was greater than that of the control plants, and HLB symptoms in some transgenic plants (RIN4-O5 and RIN4-O2) appeared earlier and severer than in the non-transgenic control plants. These results indicated that RIN4 overexpression might facilitate the *C*Las infection and disease symptom development in citrus.

## 4. Materials and Methods

### 4.1. Plant Materials

All of the plant materials used in this study were provided by the Institute of Fruit Tree Research, Guangdong Academy of Agricultural Sciences. For gene cloning, leaves of *Citrus clementina* were used. Roots, stems, leaves and flowers of sweet orange were used for the quantitative real-time PCR (qRT-PCR). Explants of *C. maxima* cv. ‘Shatianyou’ plants were used for genetic transformation. *C*Las-infected red tangerine branches were used for HLB inoculations of non-transgenic and transgenic plants.

### 4.2. Gene Cloning and Sequence Characterization

The HLB-induced *RIN4* gene’s (Ciclev10021726m) coding sequence was downloaded from the *C. Clementina* genome (https://phytozome.jgi.doe.gov/pz/portal.html#!info?alias=Org_Cclementina, accessed on 1 April 2013). Primers for gene cloning were designed using the downloaded sequences. RNA was extracted from *C. clementina* leaves using a polysaccharide polyphenol plant total RNA extraction kit (Guangzhou Xueyou Biology Co., Ltd., Guangzhou, China) and was then used for cDNA synthesis using the PrimeScript™ II 1st-strand cDNA Synthesis Kit (TaKaRa, Dalian, China). The gene was amplified on an ABI thermal cycler using *C. clementina* cDNA as the template with RIN4F/RIN4R primers (Table 1). The 20 μL PCR reaction system contained 1 μL template cDNA, 2 μL 10 × LA PCR buffer, 1.5 μL of 2.5 mM dNTPs, 0.4 μL RIN4F/RIN4R primers, 0.2 μL LA DNA Taq polymerase (5 U/μL) and 15.5 μL ddH_2_O. The PCR amplification reaction was set as follows: pre-denaturation at 94 °C for 5 min, 35 cycles of 94 °C for 30 s, 60 °C for 35 s and 72 °C for 45 s, and final extension at 72 °C for 7 min. The PCR products were electrophoresed on a 2% agarose gel, and the target fragment was purified using AxyPrep™ DNA Gel Extraction Kit (Aishin Biotech Co., Ltd., Suzhou, China), ligated into the pEASY-T vector (Transgene, Beijing, China), and transformed into Trans1-T1 *Escherichia coli* competent cells. After PCR confirmation, the plasmids were extracted using an AxyPrep™ Plasmid Miniprep Kit Nucleic Acid Purification Kit and sent to the Beijing Liuhe Huada Gene Technology Co., Ltd. (Beijing, China). for sequencing validation. The correct plasmid was then used for vector construction. Primers for overexpression-vector and subcellular-localization vector construction, as well as qRT-PCR analysis, were designed using the obtained gene sequence. Information of the primers used in this study are listed in Table 1.

### 4.3. Sequence Analyses of RIN4 and Its Corresponding Protein and Promoter

The RIN4 nucleotide sequence and predicted amino acid sequence were analyzed using NCBI Blast (http://blast.ncbi.nlm.nih.gov/Blast.cgi, accessed on 2 April 2013). Multiple alignments were conducted using DNAMAN5.2.2 software. For the physicochemical property analysis, ProtParam (http://web.expasy.org/protparam, accessed on 2 April 2013) was used. Signal peptides and transmembrane domains were analyzed using InterProScan (http://www.ebi.ac.uk/Tools/pfa/iprscan/, accessed on 2 April 2013). The conserved domain of RIN4 protein was predicted using CDD (https://www.ncbi.nlm.nih.gov/Structure/cdd/wrpsb.cgi, accessed on 2 April 2013). NetPhos 3.1 Server (http://www.cbs.dtu.dk/services/NetPhos/, accessed on 2 April 2013) was used for the posttranscriptional modification prediction of citrus RIN4. The upstream 2000 bp sequence of the *RIN4* gene (Ciclev10021726m) was downloaded from the *C. clementina* genome, and *cis*-acting elements were analyzed using the PlantCARE (http://bioinformatics.psb.ugent.be/webtools/plantcare/html/, accessed on 1 May 2019).

### 4.4. qRT-PCR Analysis of RIN4 Expression under Different Treatments

Because the cloned *RIN4* sequences of *C. clementina* and sweet orange are the same, to investigate the organ expression pattern of *RIN4* in citrus, sweet orange leaf, stem, root and flower samples were used. According to the organ expression level result, we further confirmed the HLB-inducible pattern of *RIN4* in leaves of *C*Las-infected citrus plants. Ten one-year-old red tangerine seedlings were divided into two groups. One group was grafted with two *C*Las-infected red tangerine buds and the other group was grafted with two healthy red tangerine buds as a control. DNA was extracted and used as the template for the detection of *C*Las using PCR two months after grafting [43]. The *C*Las detection primer sequences were: HLB- forward (F): GCG CGT ATG CAA TAC GAG CGG CA and HLB-reverse (R): GCC TCG CGA CTT CGC AAC CCA T, and produced a 1160-bp fragment. Red tangerine leaves that were PCR positive and control leaves were used for RNA extraction and further qRT-PCR analysis using the primer pair RIN4rF/RIN4rR (Table 1).

Samples from three-year-old potted sweet oranges were used for the expression pattern analysis of *RIN4* under different phytohormone and stress treatments. For the gibberellic acid (GA), indole-3-acetic acid (IAA), salicylic acid (SA), jasmonic acid (JA) and abscisic acid (ABA) treatments, 100 μM phytohormone solutions were directly sprayed on the leaves of sweet oranges according to the method described by Ramamoorthy et al. [58]. Leaf samples were taken at 0, 2, 4, 8 and 16 h after treatment. For the salt treatment, soil was thoroughly watered using a 250 mM NaCl solution. Leaves were taken at 0, 2, 4, 8 and 16 h after treatment. To mimic drought, a 30% PEG solution was used to water the soil thoroughly, and leaf samples were taken at 0, 0.5, 1, 2, 3 and 4 h after treatment. For the wounding treatment, leaves were injured using needle-nose pliers to produce a visible indentation, and leaf samples were harvested at 0 min, 10 min, 0.5 h, 4 h and 24 h after treatment. All of the leaf samples were quickly frozen in liquid nitrogen after harvesting and stored in a refrigerator at −80°C for later use in qRT-PCR experiments.

qRT-PCR reactions were performed according to the protocol of the SYBR^®^ Premix Ex Taq™ II (Perfect Real Time) kit (TaKaRa). The 20 μL reactions included 10 μL 2× SYBR^®^ Premix Ex Taq, 1.0 μL cDNA, 0.4 μL of each RIN4rF/RIN4rR primer and 8.2 μL double-distilled water. The amplification procedure was as follows: pre-denaturation at 95 °C for 30 s, followed by 40 cycles of 95 °C for 5 s and 60 °C for 20 s. For melting curve analysis, the fluorescence intensity data were collected in the range of 75~95 °C with a rate of 0.2 °C per 10 s at the end of the run. qRT-PCR was carried out in Lightcycler480II (Roche, Switzerland) using SYBR Green real time PCR Master Mix (Toyobo, Osaka, Japan) according to Cheng et al. [59]. *β-actin* gene was used as the endogenous control. The expression was calculated by using 2^−ΔΔCt^ method and normalized against *β-actin* expression level. Three biological and technical replicates were performed for each sample under each treatment. The results were shown as mean ± standard deviation (SD) from nonuplicate experiments. The significance of gene expression difference was calculated by using SPSS version 19.

### 4.5. Vector Construction

The *RIN4*-overexpression vector was constructed as described in our previous study [38]. Briefly, the pEASY-T vector carrying the *RIN4* gene was digested with *Asc*I and *Sma*I, ligated into the pFGC5941 vector and transformed into *E. coli*. After being confirmed to be positive by PCR, plasmids for overexpression were purified and transformed into *Agrobacterium tumefaciens* EHA105 electroporation competent cells [60]. The PCR-verified positive clones were kept at −80 °C for further use.

To construct the subcellular-localization vector of RIN4, the RIN4SF/RIN4SR primer pair was used to amplify the *RIN4* sequence with *Xba*I and *Bam*HI digestion sequence by using *C. clementina* leaf cDNA as the template. The PCR products were ligated into the pEASY-T vector and then were transformed into *E. coli* Trans-T1 component cells. After plasmid purification and double digestion with *Xba*I and *Bam*HI, the target sequence was ligated into the pBEGFP vector and transformed into *E. coli*. After PCR screening, the recombined vectors were extracted and transformed into *A. tumefacien*s EHA105 electroporation competent cells. The PCR-verified positive clones were kept at −80 °C for further use.

### 4.6. Subcellular Localization Analysis

Onion epidermals from the third and the fourth layers were cut into 1 cm^2^ pieces and pre-cultured on MS medium at 23 °C for 1 day. *A. tumefaciens* clone carrying the pBEGFP-RIN4 plasmid was selected and cultivated in YEB solution at 28 °C, 220 rpm for about 2 days, centrifuged at 4500 rpm for 10 min and re-suspended in YEB liquid media supplemented with 10 mM MgCl_2_ and 20 μM acetosyringone to OD600 of 0.5 to 0.7. The pre-cultured onion epidermal cells were immersed in the agroinfiltration for 30 min and cultivated on MS medium for 36 h. Then, the subcellular distribution of the green fluorescence protein (GFP) fluorescence was observed according to the method described by Bai et al. [61]. To determine whether it was located in the cell membrane or cell wall, a 30%-sucrose solution was used for the induction of plasmolysis. Photos were taken before and after the plasmolysis experiment. *A. tumefaciens* carrying the empty pBEGFP vector was used as the control.

### 4.7. Citrus Transformation and HLB Resistance Evaluation

Citrus transformations were performed using *C. maxima* cv. ‘Shatianyou’ epicotyls as explants [59,62]. When the candidate resistant plants had five to eight leaves, genomic DNA was isolated from fully expanded leaves and then used for the PCR-based identification of transgenic plants. The DNA was used as the template to amplify *35S* and *BAR* genes with the gene-specific primers, 35Sf/35Sr (35Sf: TCA TAA ACC AAG GCA AGT AAT AGA G; 35Sr: GAT AGT GGG ATT GTG CGT CAT; target length 546 bp) and BARf/BARr (BARf: TGC ACC ATC GTC AAC CAC TAC ATC; BARr: GCT GCC AGA AAC CCA CGT CAT; target length 433 bp), respectively. PCR-positive transgenic plants were multiplied by grafting their buds onto red tangerine rootstocks. The graft-propagated transgenic plants were reexamined using 35Sf/35Sr and BARf/BARr primers to reduce the pseudopositive and chimera ratios [60,62]. Healthy non-transgenic ‘Shatianyou’ pomelo buds were graft-propagated and used as controls in further studies. The expression levels of *RIN4* in transgenic plants were studied by qRT-PCR using the method and procedures described in Section 4.4.

After three months, grafted transgenic and control pomelo plants that were ~20 cm tall were subjected to HLB inoculation by grafting two *C*Las-infected red tangerine buds onto the rootstocks [38]. The phenotypes of the *C*Las-inoculated plants were observed.

Additionally, DNA was extracted every month for the PCR detection of *C*Las infection using the HLB-F/HLB-R primer pair. The quantitative Taqman PCR was used to detect the titers of *C*Las in the plants using a *C*Las 16S rDNA-based primer-probe. The procedure followed was that of Li et al. [63]. Three replicates were made for each transgenic and non-transgenic plants.

## 5. Conclusions

In summary, we cloned and characterized the HLB-inducible *RIN4* gene. As an Arabidopsis *RIN4* homologous gene, its encoded protein contains the conserved the N-NOI and C-NOI domains, the same RCS1 and RCS2 sequences as Arabidopsis RIN4, three C-terminal cysteine residues and many conserved amino acid residues required for the posttranscriptional modifications of RIN4 protein. Most *RIN4*-overexpression plants showed much higher *C*Las titters and increased HLB sensitivity, indicating that the gene’s overexpression might be helpful for the pathogen infection. Our study indicated that *RIN4* is a negative regulator of citrus HLB responses, and its upregulation in HLB diseased citrus aids the infection of *C*Las.

## Figures and Tables

**Table 1:** Sequence information of primers used in this study.

Primer	Primer Sequence (5′→3′)	Application	Digestion Site
RIN4F	*ttGGCGCGCC*ATGGCACAACGTTCACATGTAC	Overexpression vector construction	*Asc*I
RIN4R	*tccCCCGGG*TTATTTCTTGCCCCAAGGAC	Overexpression vector construction	*Sma*I
RIN4SF	ctagTCTAGAgATGGCACAACGTTCACATGTAC	Subcellular localization vector construction	*Xba*I
RIN4SR	ctagTCTAGAgTTTCTTGCCCCAAGGAC	Subcellular localization vector construction	*Bam*HI
RIN4rF	CAGTCTTGAACGCTCCCCTA	qRT-PCR	-
RIN4rR	TCGACTCTTGGATTTGGCCT	qRT-PCR	-

## Data Availability

All data are available in this article and in the supplementary file.

## Supplementary Materials

The following are available online at https://www.mdpi.com/article/10.3390/ijms23136971/s1.
