# Supplementary material for: The Upregulated Expression of the Citrus RIN4 Gene in HLB Diseased Citrus Aids Candidatus Liberibacter Asiaticus Infection
Source: Int J Mol Sci. 2022 Jun 23;23(13):6971. doi: 10.3390/ijms23136971 (PMC9266415; doi:10.3390/ijms23136971)
Supplement: Supplementary file 1 [file ijms-23-06971-s001.zip › ijms-1735476-supplementary.pdf]

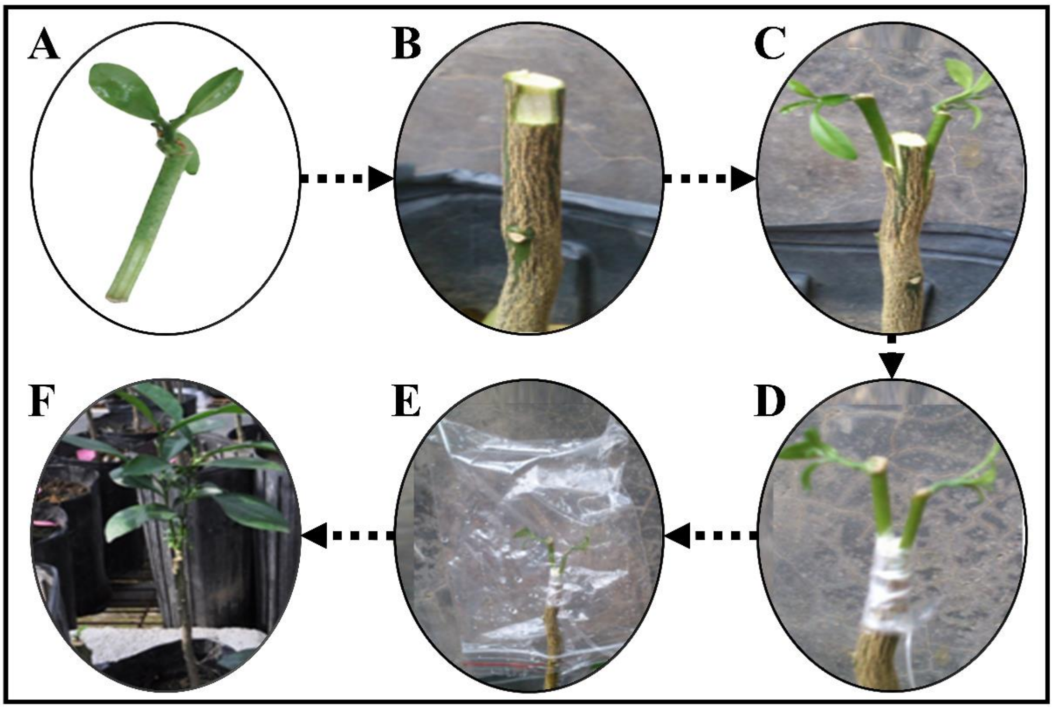

Figure S1. Grafting of the regenerated resistant buds. A: The regenerated resistant buds; B: Rootstock used for grafting; C: One or two buds were inserted into the rootstock; D: Binding; E: Bagging to maintain humidity; F: Graft-propagated transgenic plants.
